# Palmitoylethanolamide Modulates Inflammation-Associated Vascular Endothelial Growth Factor (VEGF) Signaling via the Akt/mTOR Pathway in a Selective Peroxisome Proliferator-Activated Receptor Alpha (PPAR-α)-Dependent Manner

**DOI:** 10.1371/journal.pone.0156198

**Published:** 2016-05-24

**Authors:** Giovanni Sarnelli, Alessandra D’Alessandro, Teresa Iuvone, Elena Capoccia, Stefano Gigli, Marcella Pesce, Luisa Seguella, Nicola Nobile, Giovanni Aprea, Francesco Maione, Giovanni Domenico de Palma, Rosario Cuomo, Luca Steardo, Giuseppe Esposito

**Affiliations:** 1 Department of Clinical Medicine and Surgery, University of Naples Federico II, Naples, Italy; 2 Department of Pharmacy, University of Naples Federico II, Naples, Italy; 3 Department of Physiology and Pharmacology ‘Vittorio Erspamer’, La Sapienza University of Rome, Rome, Italy; Ohio State University, UNITED STATES

## Abstract

**Background and Aim:**

Angiogenesis is emerging as a pivotal process in chronic inflammatory pathologies, promoting immune infiltration and prompting carcinogenesis. Ulcerative Colitis (UC) and Crohn’s Disease (CD) represent paradigmatic examples of intestinal chronic inflammatory conditions in which the process of neovascularization correlates with the severity and progression of the diseases. Molecules able to target the angiogenesis have thus the potential to synergistically affect the disease course. Beyond its anti-inflammatory effect, palmitoylethanolamide (PEA) is able to reduce angiogenesis in several chronic inflammatory conditions, but no data about its anti-angiogenic activity in colitis have been produced, yet.

**Methods:**

The effects of PEA on inflammation-associated angiogenesis in mice with dextran sulphate sodium (DSS)-induced colitis and in patients with UC were assessed. The release of Vascular Endothelial Growth Factor (VEGF), the hemoglobin tissue content, the expression of CD31 and of phosphatidylinositol 3-kinase/Akt/mammalian-target-of-rapamycin (mTOR) signaling axis were all evaluated in the presence of different concentrations of PEA and concomitant administration of PPAR-α and -γ antagonists.

**Results:**

Our results demonstrated that PEA, in a selective peroxisome proliferator activated receptor (PPAR)-α dependent mechanism, inhibits colitis-associated angiogenesis, decreasing VEGF release and new vessels formation. Furthermore, we demonstrated that the mTOR/Akt axis regulates, at least partly, the angiogenic process in IBD and that PEA directly affects this pathway.

**Conclusions:**

Our results suggest that PEA may improve inflammation-driven angiogenesis in colonic mucosa, thus reducing the mucosal damage and potentially affecting disease progression and the shift towards the carcinogenesis.

## Introduction

Angiogenesis is the process of new vessels development from preexisting vasculature in adult tissues and it is emerging as pivotal in the pathogenesis and progression of chronic inflammatory pathologies [1–4].

There is evidence that angiogenesis contributes to a significant dysfunction of vessel architectures, promotes the recruitment of pro-inflammatory cells, and results in a progressive loss of the epithelial integrity [5,6]. Inflammatory bowel diseases (IBD), such as Crohn’s disease (CD) and ulcerative colitis (UC) are paradigmatic examples of chronic inflammatory diseases in which angiogenesis-related factors affect diseases’ progression and severity [5–8].

A variegate class of signaling molecules/cytokines, involved in inflammation and tissue remodeling processes, co-promotes angiogenesis, such as nitric oxide (NO) or prostaglandins (PGs), but a prominent role has been identified for Vascular Endothelial Growth Factor A (VEGF). This mediator, through the activation of a complex signaling network, yields to neovascularization, worsening tissue damage and promoting the carcinogenic drift [9–10]. In keeping with this, the inhibition of angiogenetic process may represent a potential therapeutic target in IBDs, acting on both inflammation and carcinogenic risk [11,12].

Although the release of VEGF is regulated by different molecular pathways, the upstream activation of phosphatidylinositol 3-kinase/Akt/mammalian target of rapamycin (mTOR) signaling axis (Akt/mTOR pathway) has been recognized as pivotal in VEGF-related neovascularization. Indeed, the activation of this pathway determines also the overexpression of the Hypoxia-Inducible Factor (HIF)-1α, a specific transcriptional factor, which, in turn, further increases the release of VEGF [12,13]. This complex network, is physiologically induced by hypoxia in order to guarantee the appropriate tissue oxygenation, stimulating vessels formation, however a pathological over-activation of this pathway has been also described in different inflammatory diseases and several tumors [14,15].

Palmitoylethanolamide (PEA) is an N-acylethanolamide (NAE), structurally and functionally related to anandamide (AEA), with anti-inflammatory and analgesic activities. The anti-inflammatory effect of PEA depends on its ability to activate peroxisome proliferator activated receptor (PPAR)-α, a member of nuclear hormone receptor superfamily of ligand activated transcription factors [16,17]. In both mice and human colitis, PEA has been reported to decrease the release of several pro-inflammatory cytokines [18–20], and there are data suggesting that PEA also exerts a significant anti-angiogenic activity in other chronic inflammatory conditions [21, 22]. However, we recently demonstrated that PEA is able to directly reduce the release of pro-angiogenic factors in an “*in vitro*” model of colon cancer cells [23]. The potential anti-angiogenic activity of PEA during colitis has never been reported, yet. With the present study we aimed to evaluate the ability of PEA to reduce the inflammation-related angiogenesis in the colon of mice with dextran sulphate sodium (DSS)-induced colitis and in UC patients, and to characterize its mechanisms of action.

## Materials and Methods

### Animals and experimental design

Six-weeks-old wild-type (WT) male CD-1 mice (Harlan Laboratories, Udine, Italy) were used for experiments. All procedures on mice were approved by La Sapienza University's Ethics Committee. Animal care was in compliance with the IASP and European Community (EC L358/1 18/12/86) guidelines on the use and protection of animals in experimental research. Animals were randomly divided into six groups (n = 10 per group): non-colitic control group; colitic group; colitic group receiving PEA 2 and 10 mg/kg, [24, 25]; colitic group receiving PEA (10 mg/kg) and selective PPAR-α antagonist MK866 (10 mg/kg); colitic group receiving PEA and selective PPAR-γ antagonist GW9662 (1 mg/kg) [26]. Two internal control groups (n = 5 per group) were also considered: colitic group receiving PPAR-α or PPAR-γ antagonist; non-colitic group receiving daily PEA 10 mg/kg. Immunohistochemistry was performed on five groups, excluding colitic group receiving the lowest dosage of PEA.

Colitis was induced by administrating 4% DSS (MP Biomedicals, Solon, Ohio, USA) in drinking water for six consecutive days. PEA alone, or combined with PPAR antagonists, was given by intraperitoneal administration from day 2 to 6 and then animals were sacrificed at day 7 by carbon dioxide inhalation followed by cervical dislocation. Colons were isolated to perform histochemical and biochemical analyses as described below.

In order to further confirm the involvement of PPAR-α in mediating the effects of PEA, additional sets of experiment were conducted in six-weeks-old wild-type PPAR-α null (KO) mice (Taconic, Germantown, New York, USA), that were divided in the following groups: vehicle; colitic; colitic receiving daily PEA 10, 50 or 100 mg/kg, n = 5 for each group, respectively.

### Cultured human intestinal biopsies

The experimental group comprised 10 patients with a new diagnose of UC (4 women; age range 21–58 years; endoscopic MAYO score>2) and 5 control subjects (3 men; age range 42–60 years; absence of gastrointestinal symptoms) undergoing colonoscopy for colon cancer screening. Exclusion criteria were history of cancer, use of 5-aminosalicylic acid, immunosuppressant, anti-platelet and anti-coagulant drugs. Also patients suffering from cardiovascular, renal or respiratory comorbidities were excluded. All subjects received and signed an informed consent and the Federico II University Ethical committee approved the protocol.

Four mucosal biopsies from the sigmoid region of UC patients and two biopsies from the same site of controls were collected and cultured in FBS-supplemented Dulbecco Modified Eagle’s Medium (DMEM) at 37°C in 5% CO2/95% air. All biopsies were cultured for 24 hours, with or without PEA at the following concentrations 0,001, 0,01, 0,1 μM [27]. Biopsies were then homogenized and analyzed by western blot as described below. PFA-fixed samples were used for immunohistochemistry, this analysis was only performed on biopsies stimulated with the highest dosage of PEA (0,1 μM).

### Protein extraction and western blot analysis

Mice and human specimens were homogenized in ice-cold hypotonic lysis buffer and protein concentration was determined using Bio-Rad protein assay kit (Bio-Rad, Milan, Italy). Analysis of total Akt, phosphor-Akt, total mTOR, phosphor-mTOR, total p70S6K, phosphor-p70S6K, anti-VEGF-R, anti-EGF receptor, anti-HIF1α and β-actin protein expression was performed on total protein fractions of homogenates. Equivalent amounts of homogenates (50 μg) underwent electrophoresis through a polyacrilamide minigel. Proteins were then transferred onto nitrocellulose membranes that were saturated by incubation with 10% nonfat dry milk in 1× PBS overnight at 4°C and then incubated, according to the experimental protocols with: mouse anti-total Akt (1:1000 v/v, Cell signaling technology, Euroclone, Pero, MI, Italy); rabbit monoclonal anti phosphor-Akt (Ser ^473^), (1:2000 v/v, Cell signaling technology, Euroclone, Pero, MI, Italy); rabbit polyclonal anti total mTOR (1:1000, Abcam, Cambridge, UK); rabbit polyclonal anti phosphor-mTOR (pSer^2448^) (1:1000 v/v, Cell signaling technology, Euroclone, Pero, MI, Italy); rabbit polyclonal anti total p70S6K (1:1000 v/v Cell Signaling Technology, Euroclone, Pero, MI, Italy); and rabbit polyclonal anti-phosphor-p70S6K (Thr421/Ser424, Thr389); (1:1000 v/v, Cell Signaling Technology, Euroclone, Pero, MI, Italy); rabbit monoclonal anti-VEGF receptor (1:1000 v/v, Cell Signaling Technology, Euroclone, Pero, MI, Italy); rabbit polyclonal anti-EGF receptor (1:1000 v/v, Abcam, Cambridge, UK); mouse monoclonal anti-HIF1α (1:500 v/v, Sigma Aldrich, MI, Italy) and mouse anti-β-actin (1:2000 v/v, Santa Cruz Biotechnology, Santa Cruz, California, USA). Membranes were then incubated with the specific secondary antibodies conjugated to horseradish peroxidase (HRP) (Dako, Milan, Italy). Immune complexes were revealed by enhanced chemiluminescence detection reagents (Amersham Biosciences, Milan, Italy). Blots were analyzed by scanning densitometry (GS-700 imaging densitometer; Bio-Rad). Results were expressed as OD (arbitrary units; mm^2^) and normalized on the expression of the housekeeping protein β-actin.

### Enzyme-linked immunosorbent assay for VEGF and EGF

Enzyme-linked immunosorbent assay (ELISA) for VEGF (Abcam, Cambridge, UK) was carried out on mice and human specimens supernatants according to the manufacturer's protocol. Absorbance was measured on a microtitre plate reader. In a subset of experiments an ELISA for EGF (Abcam, Cambridge, UK) was also carried out on plasma of PPAR-α null (KO) mice; VEGF and EGF levels were thus determined using standard curves method.

### Immunohistochemistry

Mice and human specimens were fixed in buffered formalin, embedded in paraffin and cut into 5μm-thick serial sections. According to manufacturer’s instructions, after heat-mediated antigen retrieval, the tissue was formaldehyde fixed and blocked with serum. The tissue was incubated with the primary antibody anti CD31 (1:50 v/v, Abcam, Cambridge, UK) for 20 minutes. After three 5-min washes, the secondary antibody was added and the samples were incubated at room temperature for 20 min. The streptavidin-HRP detection system (Chemicon Int., Temecula, CA, USA) was added and samples were incubated at room temperature. After three 5-min washes, 50 μL of chromogen was added and the reaction terminated after 1 min in water. Sections were then counterstained with haematoxylin eosin at room temperature. Negative controls were performed by omitting primary antibody. Slides were thus analyzed with a microscope (Nikon Eclipse 80i by Nikon Instruments Europe), and images were captured at 20X magnification by a high-resolution digital camera (Nikon Digital Sight DS-U1). The amount of vascularization in each colon section was quantified as a percentage of tissue area immunopositive for CD31 at a 20X magnification in 6 regions, each amounting to a 0,20- mm^2^ area, and expressed as vessel density of (%).

### Haemoglobin content measurement

As previously demonstrated, hemoglobin content measurement represents an appropriate method for the detection and quantification of angiogenesis in tissues [28,29]. Mice and human colonic specimens were collected and weighted, samples were then homogenized in 1 × PBS. After centrifugation at 2500 × g for 20 min at 4°C, the supernatants were further centrifuged at 5000 × g for 30 min, and hemoglobin concentration in the supernatant was determined spectrophotometrically at 450 nm by the hemoglobin assay kit (Sigma Aldrich, MI, Italy). Values were expressed as mg haemoglobin/g of wet weight.

### Statistical analysis

Results were expressed as mean±SD of n experiments. Statistical analysis was performed using parametric one-way analysis of variance (ANOVA) and multiple comparisons were performed by Bonferroni’s posthoc test; p values <0.05 were considered significant.

## Results

### Mice DSS-induced colitis is associated with an increase of angiogenesis that is inhibited by Palmitoylethanolamide

In mice with DSS-induced colitis, bloody diarrhea together with loss of body weight and increase of spleen size were observed from day 4 until the sacrifice. As expected, the immune infiltrate and the inflammatory mediators (NO, PGE_2_ and TNFα) were also significantly increased in treated mice (data not shown).

**Fig 1:**
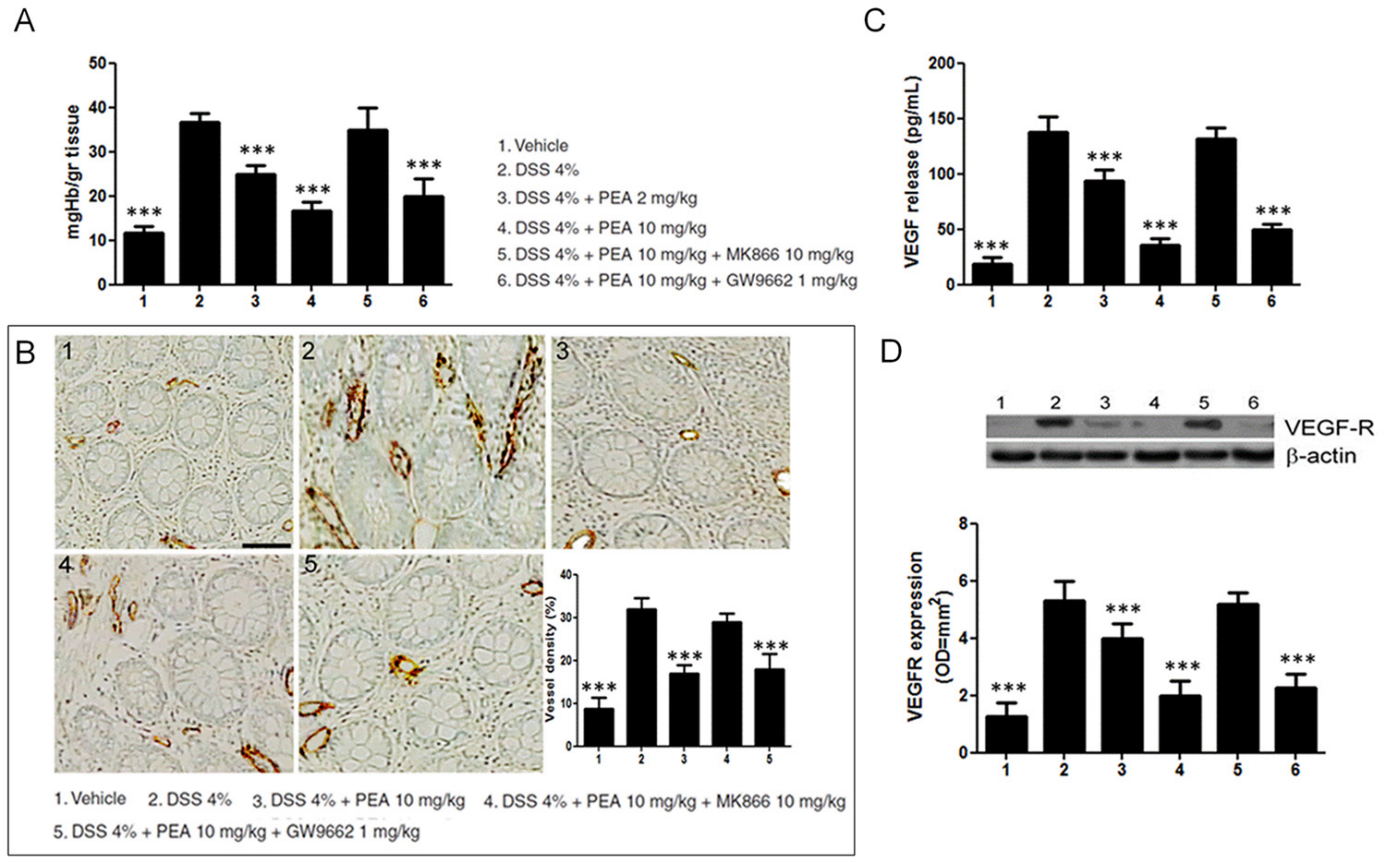
Palmitoylethanolamide (PEA) inhibits colitis-associated angiogenesis in mice. **(A)** DSS-induced colitis caused a significant increase of Hb-content in colonic mucosa, PEA is able to reduce, in a dose-dependent fashion, the Hb-content in colitis mice; this effect persisted in presence of PPARγ antagonist (GW9662) while it was nullified by PPARα antagonist (MK866). (**B)** Immunohistochemical images showing the expression of CD31 on untreated mice colonic mucosa **(panel 1)**, DSS-treated mice colonic mucosa **(panel 2)**, DSS-treated mice colonic mucosa in presence of PEA (10 mg/Kg) alone **(panel 3)**, PEA (10 mg/Kg) plus MK866 10 mg/Kg **(panel 4)**, and PEA (10 mg/Kg) plus GW9662 1 mg/Kg **(panel 5)**. Magnification 20X; scale bar: 100μm. The graph summarizes the relative quantification of CD31 expression (%) on mice colonic mucosa in the same experimental groups, showing the reduction of CD31 expression in colitic mice after PEA administration, except for the group also treated with the antagonist of PPARα. (**C)** VEGF release resulted increase in DSS-treated mice and it was significantly reduced by PEA treatment in a PPARα dependent manner. (**D)** Western blot analysis and relative densitometric analysis (arbitrary units normalized on the expression of housekeeping protein β-actin) of VEGF-receptor (VEGF-R) expression, showing similar results to VEGF release. Results are expressed as mean±SD. *p<0.05, **p<0.01 and ***p<0.001 versus DSS-treated mice.

Hemoglobin tissue content and the expression of CD31, a blood vessel endothelial marker, were evaluated to detect the effect of PEA on angiogenesis. The hemoglobin content was significantly increased in mice with DSS-induced colitis compared to controls (36,6±2 vs 11,6±1,6 mgHb/gr tissue; p<0,0001), but such increase was significantly reduced in mice receiving PEA (2 and 10 mg/kg) in a dose-dependent fashion (-36% and -60%, respectively; all p<0,001, Fig 1A). Co-administration of the PPAR-α antagonist, MK866, but not PPAR-γ antagonist, GW966, significantly reverted the effects of PEA on hemoglobin content, likely indicating the selective involvement of PPAR-α (Fig 1A).

A significant higher density of CD31 positive cells was also observed in the inflamed mucosa of DSS-treated mice compared to controls, and, in line with the above described results, this was significantly inhibited by PEA in PPAR-α dependent mechanism (Fig 1B).

In order to evaluate whether VEGF regulates the inflammatory-related angiogenesis in mice colitis and if PEA may directly affect this specific pathway, we assessed the release of VEGF and the expression of its receptor (VEGF-R). As expected, in DSS-treated mice the release of VEGF was significantly higher than in controls, and this was associated with an increased expression of VEGF-R (18,7±6,2 vs 137,5±14 pg/mL and 1,25±0,5 vs 5,27±0,7 OD*mm^2^, respectively; all p<0,0001; Fig 1C and 1D). Again, the administration of PEA (2 and 10 mg) significantly inhibited the release of VEGF and the expression of its receptor in a dose-dependent manner (-32 and -72%, -33 and -66% vs DSS-treated mice, respectively; all p<0,001, Fig 1C and 1D), and this effect was significantly affected by MK866, but not by GW9662, co-administration (Fig 1C and 1D).

**Fig 2:**
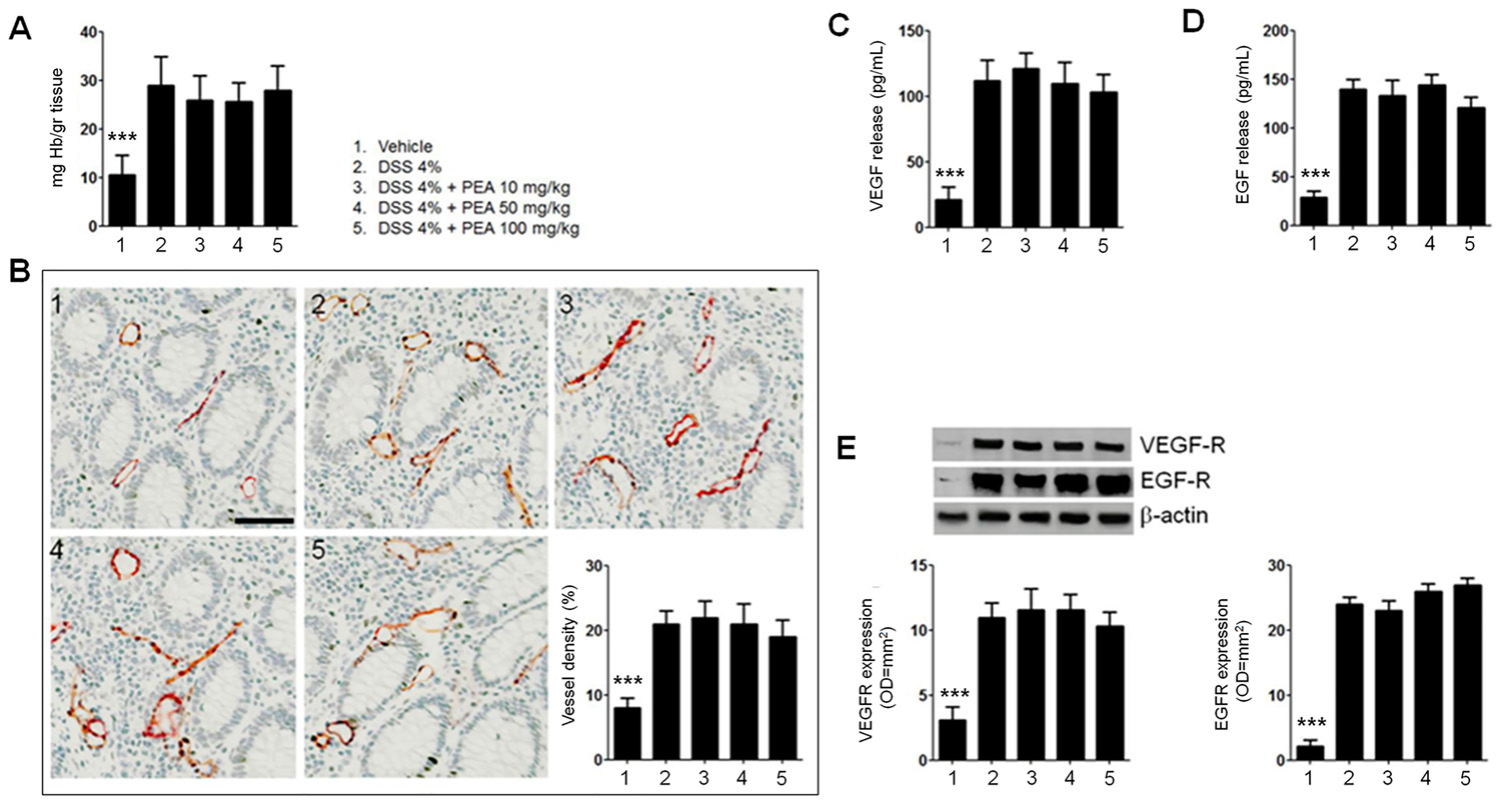
PEA had no effects on colitis-associated angiogenesis in PPARα null (KO) mice. **(A)** DSS-induced colitis caused a significant increase of Hb-content in colonic mucosa compared to untreated group, PEA at different concentrations (10–100 mg/kg) did not show any effect on the Hb-content in colitis PPARα (KO) mice. **(B)** Immunohistochemical images showing the expression of CD31 on untreated (panel 1), DSS-treated (panel 2), and DSS-treated mice in presence of PEA (10 mg/Kg) (panel 3), PEA (50 mg/Kg) (panel 4) and PEA (100 mg/Kg) (panel 5). Magnification 20X; scale bar: 100μm. The graph summarizes the relative quantification of CD31 expression on mice colonic mucosa in each respective group of mice, and shows the lack of any significant effect of PEA in reducing CD31 expression in colitic PPARα null mice. In PPARα null mice, DSS-treatment significantly increased the release of VEGF (C) and EGF (D) and these were unchanged by PEA administration, regardless of the concentrations used. (E) Western blot analysis and relative densitometric analysis (arbitrary units normalized on the expression of housekeeping protein β-actin) of VEGF-receptor (VEGF-R) and EGF-receptor (EGF-R) expression, showing similar results to VEGF and EGF release. Results are expressed as mean±SD. ***p<0.001 versus untreated mice.

In Fig 2 it is summarized that PEA failed to induce any significant effect in DSS-treated PPAR-α null mice, further supporting the specific involvement of PPAR-α.

### Palmitoylethanolamide inhibits the angiogenesis in the mucosa of patients with UC

In order to verify the effect of PEA in the context of human colonic inflammation we performed the same experimental protocol on mucosal biopsies from UC patients. As previously reported [6,28,29], in the mucosa of UC patients the concentration of hemoglobin was higher than in controls (43±6 vs 11,8±3 mgHb/g tissue p<0,001, Fig 2A) and challenge with PEA induced a significant reduction of at hemoglobin content, in a dose dependent manner (-33, -55 and -67%, for 0,001, 0,01 and 0,1 μM respectively; all p<0.01, Fig 2A). Similarly to what observed in the inflamed colon of mice, 0,1 μM of PEA were also able to significantly reduce the number of CD31 positive cells in the mucosa of patients with UC (-50%; p<0,001, Fig 2B).

As compared to control biopsies, the release of VEGF and the expression of its receptor were also significantly increased in the mucosa from UC patients (191,6±12 vs 41,6±8 pg/mL and 10±0,8 vs 0,5±0,1 OD*mm^2^, respectively; all p<0,001, Fig 2C and 2D). Again, the challenge with PEA was demonstrated to induce a significant and dose-dependent reduction of both VEGF release and VEGFR expression (-37, -53 and -70%, and -30, -50 and -70%, for 0,001, 0,01 and 0,1 μM respectively; all p<0,001, Fig 2C and 2D). Similarly to what observed in the mice all the above described effects of PEA were dependent by PPAR-α since co-administration of MK866, but not of GW9662 significantly inhibited its effects even at the highest dose (Fig 2A–2D)

### The anti-angiogenic effect of PEA depend upon Akt/mTOR/p70S6K and HIF-1α pathways downregulation

**Fig 3:**
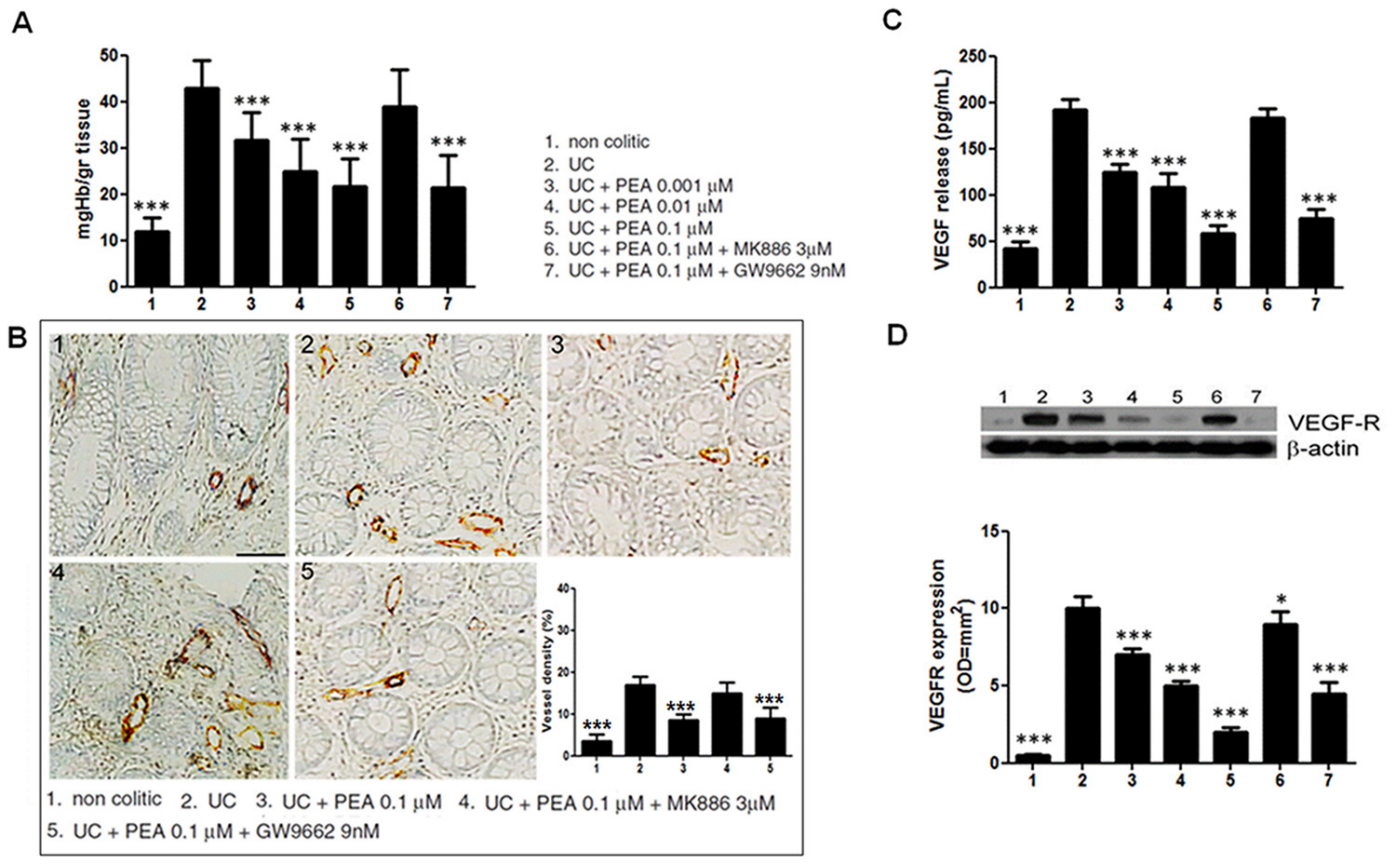
Effects of palmitoylethanolamide (PEA) on molecular markers of angiogenesis in human. **(A)** Ulcerative colitis caused a significant increase of Hb-content in colonic mucosa, PEA is able to reduce, in a dose-dependent fashion, the Hb-content in colitis patients; this effect persisted in presence of PPARγ antagonist (GW9662) while it was nullified by PPARα antagonist (MK866). (**B)** Immunohistochemical images showing the expression of CD31 on: colonic mucosa of controls **(panel 1)**, UC patients colonic mucosa **(panel 2)**, UC patients colonic mucosa in presence of PEA (0,1 μM) alone **(panel 3)**, PEA (0,1 μM) plus MK866 3 μM **(panel 4)**, and PEA (0,1 μM) plus GW9662 (9nM) **(panel 5)**. Magnification 20X; scale bar: 100μm. The graph summarizes the relative quantification of CD31 expression (%) on human colonic mucosa in the same experimental groups, and, as described in mice, PEA administration caused a significant reduction of CD31 expression, except after co-administration of PPARα antagonist. **(C)** VEGF release resulted increase in uncreative colitis patients and it was significantly reduced by PEA treatment in a PPARα dependent manner **D)** Western blot analysis and relative densitometric analysis (arbitrary units normalized on the expression of housekeeping protein β-actin) of VEGF-receptor (VEGF-R) expression, showing a similar behavior to VEGF release. Results are expressed as mean±SD. *p<0.05, **p<0.01 and ***p<0.001 versus untreated biopsies from ulcerative colitis patients.

In order to verify whether PEA has a direct anti-angiogenic effect or this effect was related to its anti-inflammatory properties, we evaluated the Akt/mTOR/p70S6K pathway, that is directly involved in the angiogenesis. In the inflamed colon of DSS-treated mice phosphor-Akt, phosphor-mTOR and p70S6K- phosphorylation, were significantly up-regulated, and all were dose-dependently and significantly reduced by intraperitoneal administration of PEA (Fig 3A and 3B). The expression of the hypoxia-Inducible Factor (HIF)-1α was also significantly reduced by -43,3 and -64% after treatment with PEA at 2 and 10 mg/kg, respectively (Fig 3A and 3B).

Similarly to what observed in the mouse colon, the expression of phosphor-Akt, -mTOR, -p70S6K and HIF-1α were all significantly overexpressed in the mucosa of UC patients (14,7±1,5 vs 1,4±0,7, 13±1 vs 1,4±0,3, 12±0,8 vs 1±0,4 and 8,5±2,7 vs 0,4±0,1 OD = mm^2^, respectively vs. control; all p<0,001; Fig 3C and 3D), and they were significantly reduced by PEA in a dose-dependent fashion (all p<0,001, Fig 3C and 3D).

**Fig 4:**
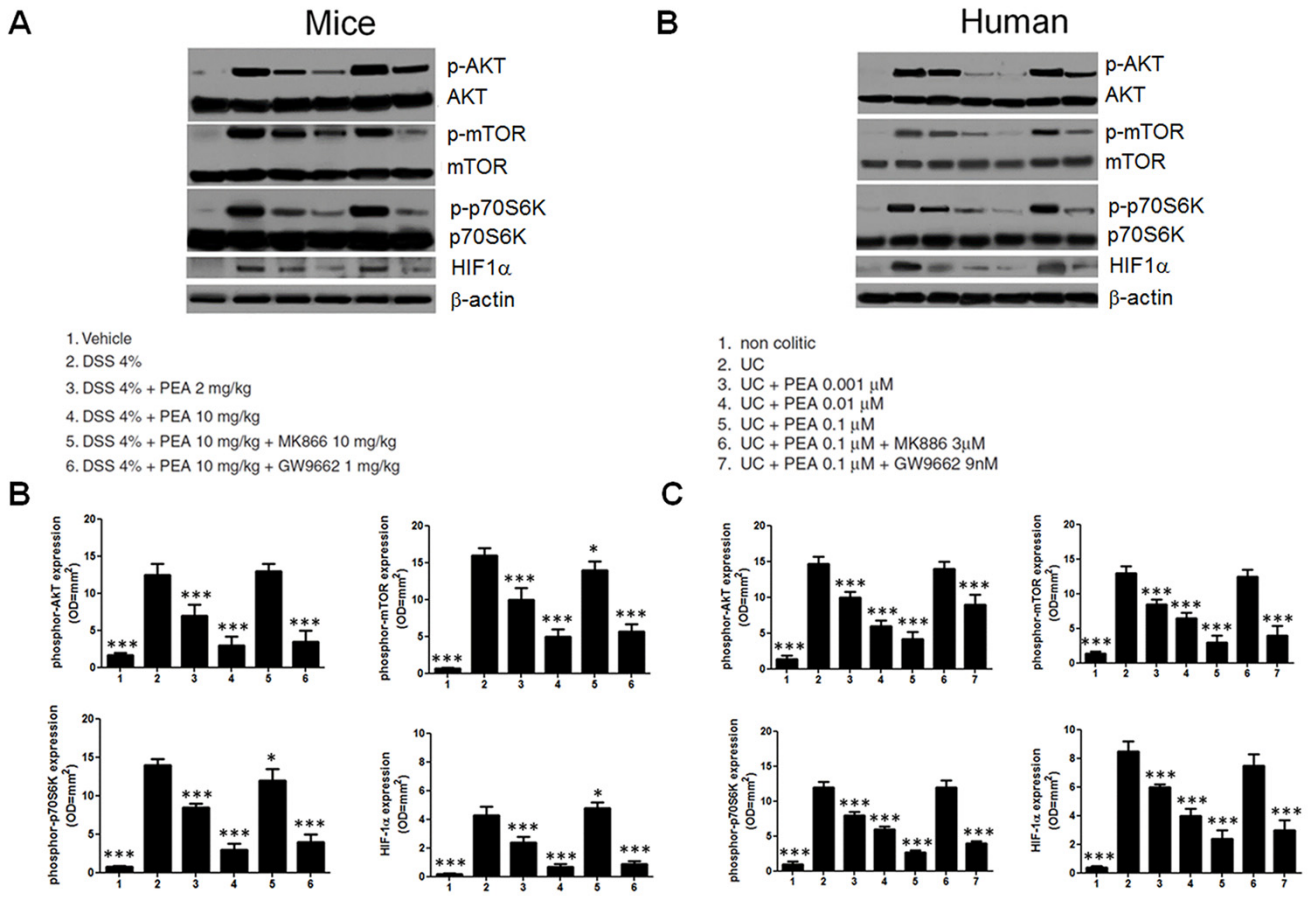
Effects of palmitoylethanolamide (PEA) on Akt/mTOR/p70S6K axis activation and HIF-1α expression in DSS-induced colitis and in ulcerative colitis. **(A)** Western Blot analysis and **(B)** relative densitometric analysis showing the effects of PEA (2 mg/Kg and 10 mg/Kg), given alone or in the presence of MK866 (10 mg/Kg) or GW 9662 (1 mg/Kg), on the expression of phosphor-Akt, phosphor-mTOR, phosphor-p70S6K and HIF-1α in mice with DSS-induced colitis. Western blot analysis **(C)** and relative densitometric analysis showing the effects of PEA at increasing concentration (0.001 μM, 0.01 μM and 0.1 μM) given alone and in the presence of MK866 (3 μM) or GW9662 (9 nM), on the expression of phosphor-Akt, phosphor-mTOR, phosphor-p70S6K and HIF-1α in UC patients biopsies. In both human and mice, colitis induced the activation of pro-angiogenic Akt/mTOR/p70S6K pathway, and PEA resulted able to reduce it, in a dose-dependent and PPARα dependent fashion. Results are expressed as mean ± SD. *p<0.05, **p<0.01 and ***p<0.001 versus DSS-treated mice (A and B) or vs untreated colonic biopsies from UC patients (C and D).

**Fig 5:**
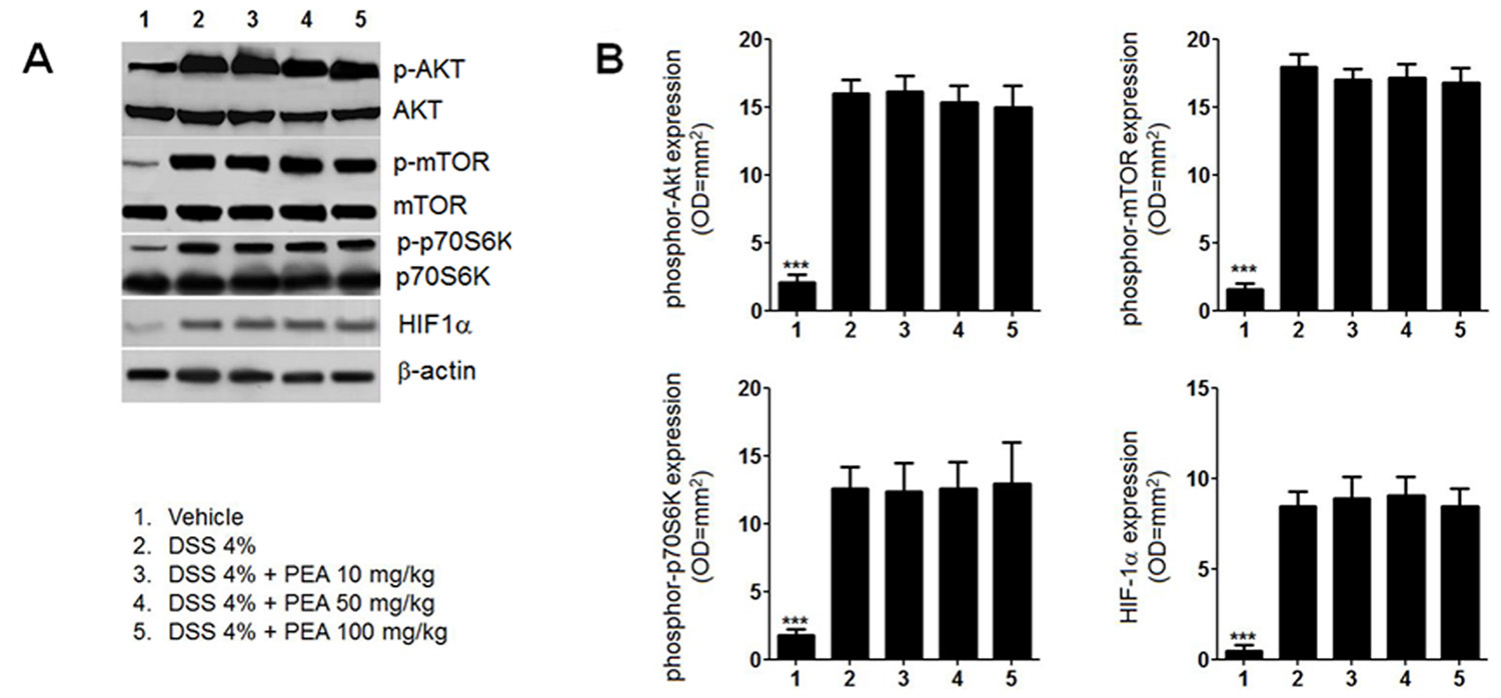
Palmitoylethanolamide (PEA) failed to significantly affect DSS-induced Akt/mTOR/p70S6K axis activation and HIF-1α expression in PPARα null (KO) mice. **(A)** Western Blot analysis and **(B)** relative densitometric analysis showing the effects of PEA (10–100 mg/kg) on the expression of phosphor-Akt, phosphor-mTOR, phosphor-p70S6K and HIF-1α in DSS-treated PPARα null (KO) mice. DSS exposure significantly induced the activation of the pro-angiogenetic Akt/mTOR/p70S6K pathway as compared to untreated mice, but treatment with PEA had no significant effect in treated mice, regardless of the concentration used. Results are expressed as mean ± SD. ***p<0.001 versus untreated mice.

In both mouse and human specimens the ability of PEA to reduce the overexpression of Akt/mTOR/p70S6K and HIF-1α pathways was significantly inhibited by concomitant administration of MK866 but not of GW9662, further supporting the concept that its effect involves the PPARα activation (Fig 4A–4D). This finding was also supported by the observation that PEA failed to significantly affect the Akt/mTOR/p70S6K and HIF-1α pathways in PPARα null mice (Fig 5).

## Discussion

Current therapies for inflammatory bowel disease are still challenging because of the relatively high rate of failure, the high costs and the risk of severe side effects due to immunosuppressive agents [30,31]. New molecules able to target different steps and pathways of the inflammatory response appear therefore a promising strategy for the treatment of these diseases.

Angiogenesis has been recently identified as a key event in the context of intestinal inflammation, whose extent significantly correlates with both the severity and the progression of the diseases [5–8], and favors the drift toward colonic carcinogenesis [32]; inhibition of angiogenesis appears thus as a synergistic and promising therapeutic strategy in IBDs [33,34].

VEGF is implicated in the regulation of the angiogenic process in sustained inflammation, and contributes to mucosal tissue remodeling, vascular permeability and leukocyte infiltration of the inflamed mucosa [35–37]. Here we demonstrated that, both in vivo and in ex vivo, PEA, in a concentration dependent manner, significantly reduced VEGF release and the expression of its receptor, in mice and human inflamed colon, respectively; this result was also associated with a significant decrease of mucosal hemoglobin content and CD31 positive vessels density.

It has been described that PEA is able to exert an antiangiogenic activity in other experimental models of chronic inflammation, likely supporting the concept that this ability is dependent by its anti-inflammatory effects [21,22,38]. However, we recently demonstrated that PEA directly reduces the release of pro-angiogenic factors in an in vitro model of colon cancer cells, through an Akt-mTOR pathway-dependent VEGF inhibition; these results suggest that the anti-angiogenic activity displayed by PEA in the inflamed colon is not solely related by its anti-inflammatory effects [23].

In our setting, we therefore investigated whether PEA-dependent VEGF signaling inhibition modulated by the AkT/mTOR axis. Different molecular pathways are involved in the angiogenesis, but the activation of AkT/mTOR axis has been specifically related to neo-vascularization in the development of inflammation-sustained colon cancer [39–41]. In particular, it induces the over-expression of HIF-1α, a transcriptional factor related to hypoxia, that cooperates with reactive oxygen species (ROS), stimulating the release of VEGF and eventually neo-angiogenesis [42–44]. Our results demonstrated that PEA, in a PPAR-α selective and concentration dependent-manner, significantly reduced the phosphorylation of Akt, mTOR and p70S6 proteins in mice colon and ex-vivo human mucosa, leading to downstream inhibition of HIF-1α with consequent inhibition of VEGF and EGF secretion and the respective receptors expression.

Remarkably, here we also showed that all the above-described pleiotropic effects of PEA are specifically related to the activation of the PPAR-α pathway. Although the role of PPAR-γ as putative site of action of anti-inflammatory and anti-cancer drugs has been specifically addressed [45,46], the importance of PPAR-α pathway is recently emerging [16,23,27]. In keeping with this, we have demonstrated that the inhibition of the mTOR/AkT axis depends on PPAR-α activation, supporting its contribution in IBD-related angiogenesis and suggesting its protective role in inflammation-associated carcinogenesis. In addition, we provide data suggesting that PEA is able to act on the process of angiogenesis by directly modulating the endothelial cell’s functioning, as demonstrated by its effect to significantly inhibit inflammatory-associated proliferation and migration of HUVEC cells (S1 Fig).

As stated, anti-angiogenetic drugs represent an intriguing approach to treat IBDs, due to the effect on both inflammation and tumorigenesis. However, the efficacy of anti-angiogenic drugs is limited by the complexity and redundancy of the molecular pathways converging in neovascularization. In this context, PEA appears as a very interesting compound, since together with its activity on the AkT/mTOR pathway, it also significantly reduces the p38/MAPK/NF-kB axis [47–49]. It has been indeed previously demonstrated that PEA is able to inhibit the NF-kB (nuclear factor kappa-light-chain-enhancer of activated B cells) pro-inflammatory pathway, determining a strong downregulation of cyclooxygenase (COX)-2 and inducible nitric oxide synthase (iNOS) expression, with a consequent reduction of prostaglandins and nitric oxide release [48, 49]. While the role of these mediators in the inflammatory process is well established, there is evidence about their involvement in neo-vascularization and tumor growth, supporting the inflammation-associated carcinogenesis assumption [50, 51]. Interestingly, besides such anti-angiogenic and anti-inflammatory activity, PEA, as the others cannabinomimetic fatty acid derivatives, also exerts an antiproliferative effect on cancer cells, supporting its protective effects in both inflammation and cancer prevention [23, 52, 53].

To date, mesalamine is the unique drug, widely used in IBD, with both anti-inflammatory and potential anti-carcinogenic proprieties [54, 55]. However, even if rare, severe side effects to this compound, such as pancreatitis and interstitial nephritis, have been described [56]. PEA is a safe drug with a well known toxicological profile and it is already available as orally administered supplement in clinical practice [57, 58].

Although further studies are needed, palmitoylethanolamide, due to its anti-inflammatory and anti-angiogenic effects, might represent a promising “food therapy” for the prevention of inflammation-associated colon cancer, and, most importantly, be part of a combined and multi-target therapy in the management of inflammation-associated angiogenesis and the potential anti-carcinogenic activity.

## Supporting Information

S1 Fig Palmitoylethanolamide reduced migration and proliferation in DSS-treated HUVEC cells. (DOCX)
