# Supplementary material for: Palmitoylethanolamide Modulates Inflammation-Associated Vascular Endothelial Growth Factor (VEGF) Signaling via the Akt/mTOR Pathway in a Selective Peroxisome Proliferator-Activated Receptor Alpha (PPAR-α)-Dependent Manner
Source: PLoS One. 2016 May 24;11(5):e0156198. doi: 10.1371/journal.pone.0156198 (PMC4878779; doi:10.1371/journal.pone.0156198)
Supplement: S1 Fig — (DOCX) [file pone.0156198.s001.docx]

**Palmitoylethanolamide modulates inflammation-associated vascular endothelial growth factor (VEGF) signaling via the Akt/mTOR pathway in a selective peroxisome proliferator-activated receptor alpha (PPAR-α)-dependent manner.**

**Supporting information**

**PEA inhibited DSS-induced proliferation and migration in cultured Human Umbilical Vein Endothelial cell culture through a PPARα selective involvement.**

In order to further evaluate and to better characterize the effect of PEA on inflammation-associated angiogenesis we tested its ability to directly modulate the endothelial cell’s functioning.

We therefore set up an in vitro model of inflammation-induced cells dysfunction in cultured Human Umbilical Vein Endothelial cells and checked the effect of PEA on cells’ proliferation and migration, as indicated below.

**Cell proliferation and wound healing assays in Human Umbilical Vein Endothelial cell culture (HUVEC).** HUVEC cells were purchased by European Collection of Authenticated Cell Cultures (ECACC), public health England, Porton Down (Salisbury, UK) and were cultured in Endothelial Cell Growth Medium (ECGM) (Lonza Basel, CH) at 37°C, 5% (v/v) CO_2_. Depending upon the experimental plan, confluent cells were seeded in 24 or 96 multiwell plates (3.125x10^4^ cells/cm^2^) and incubated for 24 hours. After this time interval, cell medium was replaced with fresh medium and cells were treated with DSS (4% w/v) according to a slightly modified previous protocol [1]. DSS treated cells were then incubated in the presence of PEA alone (0.1µM), or in the presence of PPAR-α antagonist MK866 (3µM) or PPAR-γ antagonist GW9662 (9 nM) according to our previous studies.

Cell proliferation was evaluated by performing a 3-[4,5-dimethylthiazol-2-yl]-2,5 diphenyltetrazolium bromide (MTT) assay [2]. In brief, HUVEC cells (5×10^4^) were plated in 96-well plates and allowed to adhere for 24 h in endothelial cell growth medium in a humidified atmosphere of 5% CO_2_ and 95% air at temperature of 37°C. After that, ECGM was replaced with fresh medium and the cells were exposed to DSS 4% alone or co-incubated with PEA (0.1 µM) alone or in the 3 µM MK866 or 9 nM GW9662, respectively. After 72 h, 25 µl MTT (5 mg/ml MTT in ECGM) was added to the cells and the mixture was incubated for an additional 3 h at 37°C. Subsequently, the cells were lysed and the dark blue crystals were solubilized using a 125-µl solution containing 50% N,N-dimethylformamide and 20% (w/v) sodium dodecylsulphate (pH 4.5). The optical density (OD) of each well was determined using a PerkinElmer, Inc. (Waltham, MA, USA) microplate spectrophotometer equipped with a 620-nm filter. Cell proliferation in response to treatments was calculated using the following equation: Cell proliferation at 72 h (%) = (OD treated / OD untreated) × 100.

The wound healing assay was performed according to a modified previously reported method [3]. Briefly, the cells (5×10^5^ cells/well) were plated on a six-well plate and incubated for 24 h in endothelial cell growth medium in a humidified atmosphere of 5% CO_2_ and 95% air at a temperature of 37°C. The cell layer was scratched using a 200-µl sterile pipette tip, then cells were washed with PBS three times and exposed to DSS 4% alone or co-incubated with PEA (0.1 µM) alone for 72 h, or in the presence of 3 µM MK866 or of 9 nM GW9662, respectively. The HUVEC cells were washed twice with PBS and fixed with 4% paraformaldehyde for 30 min and images were captured using a Nikon Eclipse 80 microscope equipped with a high-resolution digital camera (Nikon Digital Sight DS-U1; Nikon Instruments, Inc.). The percentage of migration was calculated by counting the number of cells that had migrated into scratched areas compared with the number of cells that had remained in the peripheral areas.

**Results**

As summarized in the figure (S1 Fig.), Huvec cells were able to invade and fully recolonize the scratched area within 72 h, with the migration being significantly increased by DSS and reduced by PEA (+38% vs. control and -59% vs. DSS, respectively, all P<0.001); cells’ treatment with DSS also caused a significant increase of proliferation that was significantly reduced by PEA (+40% vs. control and -58% vs. DSS, respectively all P<0.001). The effect of PEA on DSS-induced cells migration and proliferation rate was significantly inhibited by MK866, but not by GW9662, further supporting the role of PPAR-α in mediating its effect.

**S1 Fig. Palmitoylethanolamide (PEA) reduced migration and proliferation in DSS-treated HUVEC cells. (A)** Wound healing assay and **(B)** relative percentage quantification showing the effect of PEA on HUVEC cells migration in vitro rate at 72 h. DSS treatment significantly increased cell migration as compared to control condition, but this effect was inhibited by (0.1 µM). Similarly, in the panel C it is shown that PEA significantly reduced DSS-induced HUVEC cells proliferation rate at 72 h **(C).** All the effects of PEA were dramatically abolished in the presence of the PPARα antagonist MK866 (3 µM), but not by the PPARγ antagonist GW9662 (9 nM) co-administration. Results are expressed as mean ± SEM of n=3 experiments performed in triplicate.

*
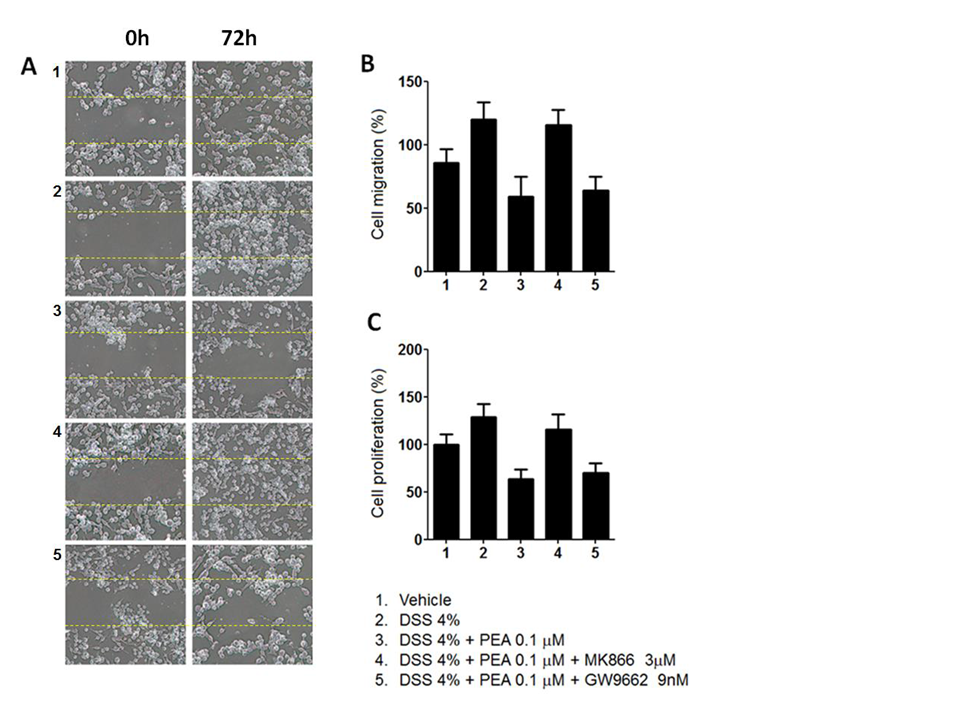
*

**References**

1. Araki Y, Sugihara H, Hattori T. In vitro effects of dextran sulfate sodium on a Caco-2 cell line and plausible mechanisms for dextran sulfate sodium-induced colitis. Oncol Rep. 2006;16 (6): 1357-62.
2. Mosmann T. Rapid colorimetric assay for cellular growth and survival: application to proliferation and cytotoxicity assays. J Immunol Methods. 1983 16; 65(1-2): 55-63.
3. Renault-Mihara F, Beuvon F, Iturrioz X, Canton B, De Bouard S, Léonard N, et al. Phosphoprotein enriched in astrocytes-15 kDa expression inhibits astrocytes migration by a protein kinase C delta-dependent mechanism. Mol Biol Cell. 2006; 17 (12): 5141-52.
